# Role of genetic mutations in folate-related enzyme genes on Male Infertility

**DOI:** 10.1038/srep15548

**Published:** 2015-11-09

**Authors:** Kang Liu, Ruizhe Zhao, Min Shen, Jiaxin Ye, Xiao Li, Yuan Huang, Lixin Hua, Zengjun Wang, Jie Li

**Affiliations:** 1 Department of Urology, The First Affiliated Hospital of Nanjing Medical University, Nanjing, China

## Abstract

Several studies showed that the genetic mutations in the folate-related enzyme genes might be associated with male infertility; however, the results were still inconsistent. We performed a meta-analysis with trial sequential analysis to investigate the associations between the MTHFR C677T, MTHFR A1298C, MTR A2756G, MTRR A66G mutations and the MTHFR haplotype with the risk of male infertility. Overall, a total of 37 studies were selected. Our meta-analysis showed that the MTHFR C677T mutation was a risk factor for male infertility in both azoospermia and oligoasthenoteratozoospermia patients, especially in Asian population. Men carrying the MTHFR TC haplotype were most liable to suffer infertility while those with CC haplotype had lowest risk. On the other hand, the MTHFR A1298C mutation was not related to male infertility. MTR A2756G and MTRR A66G were potential candidates in the pathogenesis of male infertility, but more case-control studies were required to avoid false-positive outcomes. All of these results were confirmed by the trial sequential analysis. Finally, our meta-analysis with trial sequential analysis proved that the genetic mutations in the folate-related enzyme genes played a significant role in male infertility.

Infertility is a global health dilemma and a multifactorial disorder affecting approximately 10–15% of all couples. Half of these cases are estimated to be due to male factors^1^,^2^. Nearly 8% of men of reproductive age seek medical counseling for infertility-related problems^3^. Male infertility mainly presents as defective spermatogenesis, and the causes can partly be attributed to neurogenic factors, genital tumors, germ cell aplasia, defective sperm transport, varicocele or environmental toxins^4^; however, although modern diagnostics and a large body of researches have explained the pathogenesis of male infertility, approximately 50% of infertility cases are still unaccounted for^5^,^6^,^7^. Recently, many studies analyzed genetic mutations in folate-related enzyme genes as these might be connected with male infertility.

Folates are a group of inter-convertible co-enzymes that play essential roles in DNA synthesis, methylation reactions and protein synthesis. Folate deficiency may impair the function of these metabolic pathways and result in homocysteine (Hcy) accumulation, which further leads to excessive oxidative stress and chaotic methylation reactions. This series of processes is involved in a variety of diseases, including male infertility^8^,^9^. Methylenetetrahydrofolate reductase (MTHFR), methionine synthase (MTR) and methionine synthase reductase (MTRR) are three key enzymes of the homocysteine and folate metabolic pathways.

The MTHFR gene is located on chromosome 1 (1p36.3), and its protein product catalyzes the reduction of methylenetetrahydrofolate (5,10-methyl THF) to methyltetrahydrofolate (5-methyl THF), which then donates a methyl group^10^,^11^. MTR, which maps to chromosomes 1q43, can catalyze the transfer of the methyl group from 5-methyl THF to homocysteine, which generates methionine and THF. MTRR catalyzes the reductive methylation of MTR, which maintains MTR in an active state during the folate cycle^12^,^13^ (Fig. 1). Therefore, it can be speculated that mutations in MTHFR, MTR and MTRR could be possible candidates for male infertility because of the vital functions of folate-related enzymes.

Recently, the associations between four single nucleotide polymorphisms (MTHFR C677T, MTHFR A1298C, MS A2756G and MTRR A66G) and male infertility were widely studied among several ethnicities using different genotyping methods^8^,^14^,^15^,^16^. Unfortunately, the results remain unclear^17^ or even contradictory^15^,^18^. To the best of our knowledge, no meta-analysis has been conducted to evaluate the associations of MS A2756G and MTRR A66G with male infertility. With regard to the MTHFR C677T mutation, Gupta N *et al.*^14^ performed a meta-analysis which included 13 articles and concluded that MTHFR C677T was strongly associated with male infertility; however, the conclusions were inconsistent with the results from a more recent meta-analysis conducted by Weiner As *et al.* in 2014 that analyzed the same number of studies^15^. Similar discordant results also occurred for the MTHFR A1298C mutation. Shen O *et al.* performed a meta-analysis and found that the A1298C mutation was capable of causing male infertility^19^; however, Wei B *et al.* held an opposite opinion^20^. A single study, particularly for studies with relatively small sample size, might be underpowered to reveal a small effect of the polymorphisms on disease risk^21^. To address this issue, we performed a meta-analysis with subgroup analyses from all eligible studies to obtain a more precise estimation of the relationships between mutations in folate-related enzyme genes and male infertility. Additionally, we analyzed the MTHFR haplotype and conducted trial sequential analysis.

## Materials and Methods

### Identification and Eligibility of Relevant Studies

We systematically collected all of the eligible literature from 01/01/2000 to 31/10/2014 by searching both the common English-language database (PubMed) and the Chinese literature databases [CNKI ( http://www.cnki.net) and WanFang ( http://www.wanfangdata.com.cn)]. The following search phrases were used: (MTHFR or MTRR or MTR or MS) and (polymorphism or mutation) and male infertility. Additional studies were identified by hand, searching the references in original articles and review articles.

### Criteria for inclusion and exclusion

The studies included in the current meta-analysis had to meet all the following criteria: (a) evaluation of the MTHFR C677T, MTHFR A1298C, MTR A2756G or MTRR A66G mutations and male infertility risk; (b) a case control design; and (c) sufficient published data for estimating an odds ratio (OR) with a 95% confidence interval (CI). The major reasons for exclusion of studies were as follows: (a) not involving male infertility research; (b) reviews and repeated literatures; (c) not providing the source of cases and controls and other essential information; and (d) not designed as a case control or cohort study.

### Data extraction

The information was carefully extracted from all of the eligible literatures independently by two investigators based on the inclusion criteria listed above. For a conflicting evaluation, a consensus was reached by discussion. The following information was collected from each literature: the first author’s name, the year of publication, country of origin, ethnicity, genotyping method, numbers of genotyped cases and controls and Hardy-Weinberg equilibrium (HWE) in the controls. The different ethnic descents were categorized as Asian, European, American or African. The genotyping methods were divided into PCR-RFLP, Taqman, allele-specific PCR, SSCP-PCR and sequencing. The quality of the studies was assessed using the Newcastle-Ottawa scale (NOS)^22^. An ultimate score of six stars or more was regarded as high-quality.

### Statistical analysis

Crude odds ratios (ORs) with their corresponding 95% CIs were used to assess the strength of associations between the four mutations in the folate-related enzyme genes and male infertility risk. The pooled ORs were performed for the allele contrast (M versus W), homozygote model (M/M versus W/W), heterozygote model (W/M versus W/W), dominant model (W/M + M/M versus W/W) and recessive model (M/M versus W/M + W/W). Heterogeneity across the studies was evaluated by the chi-square-based Q test and was considered statistically significant if P < 0.10. The pooled OR was assessed in both the fixed-effects model (the Mantel–Haenszel method)^23^ and the random-effects model (the DerSimonian and Laird methods)^24^. The fixed-effects model would be adopted when the studies were found to be homogeneous (with P > 0.10 for the Q test). Otherwise, the random-effects model would be used. To further investigate the possible sources of heterogeneity, meta-regression analysis and Galbraith plot were performed. Stratified analyses were conducted by ethnicity and sample size (subjects ≧ 500 in both case and control groups or not). Sensitivity analyses were performed to assess the stability of the results, namely, a single study in the meta-analysis was deleted each time to reflect the influence of the individual data set on the pooled OR. Publication bias was estimated with Begg’s funnel plot and Egger’s linear regression test. To adjust the values for multiple comparisons, we applied the Benjamini-Hochberg (BH) methods, which control for false discovery rate (FDR)^25^. Besides, departure from HWE in the controls was tested by the chi-square test for goodness of fit, and a P < 0.05 was considered as a significant disequilibrium. All analyses were done with Stata software (version 12.1; StataCorp LP, College Station, TX, USA). All p values were obtained by two-sided test.

### Trial sequential analysis

We examined the reliability and conclusiveness of the available evidence using a novel statistical analysis software called TSA (The Copenhagen Trial Unit, Center for Clinical Intervention Research, Denmark). In the meta-analysis, it is important to minimize the risk of making a false-positive or false-negative conclusion^26^; however, a meta-analysis may lead to type I errors and type II errors if the data are insufficient or if there is repeated testing for significance when new trials are added^27^,^28^,^29^. One solution is to adjust the thresholds for which results are considered statistically significant and which results are not. Alternatively, another one is to penalize the test statistic according to the strength of evidence and the number of performed significance tests^30^. The TSA software provides methods for both approaches. Conclusions made using TSA show the potential to be more reliable than those using traditional meta-analysis techniques. Empirical evidence suggests that the information size considerations and adjusted significance thresholds may eliminate early false positive findings due to imprecision and repeated significance testing in meta-analyses^31^,^32^. TSA can adjust the threshold for statistical significance depending on the quantified strength of evidence and the impact of multiplicity ( www.ctu.dk/tsa). To avoid an increase of overall type I error, we performed trial sequential analyses and then calculated the heterogeneity corrected optimal information size (HOIS). HOIS was estimated according to an overall type-I error of 5%, a power of 95% and a relative risk reduction (RRR) assumption of 10%. A continuity correction of 0.5 was also applied in zero-event trials.

## Results

### Characteristics of studies

**Figure 2:**
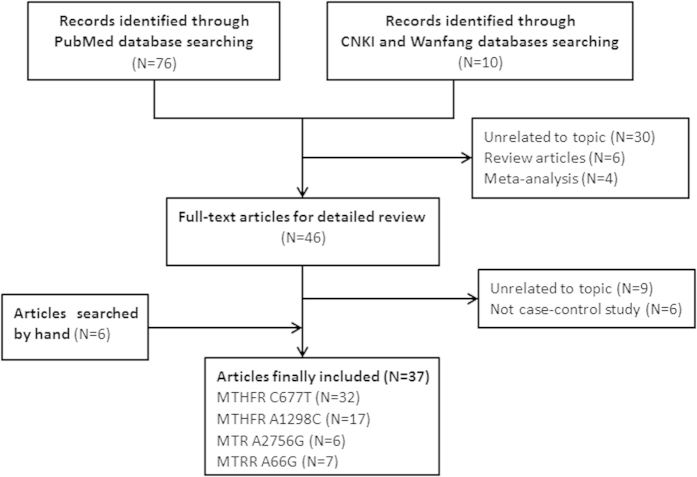
Studies identified with criteria for inclusion and exclusion.

Through the literature search and selection based on the inclusion criteria, a total of 37 papers^8^,^11^,^14^,^15^,^16^,^17^,^18^,^33^,^34^,^35^,^36^,^37^,^38^,^39^,^40^,^41^,^42^,^43^,^44^,^45^,^46^,^47^,^48^,^49^,^50^,^51^,^52^,^53^,^54^,^55^,^56^,^57^,^58^,^59^,^60^,^61^,^62^ were included in the meta-analysis (Fig. 2). The numbers of case-control studies containing the MTHFR C677T mutation, MTHFR A1298C mutation, MTR A2756G mutation and MTRR A66G mutation were 32, 17, 6 and 7, respectively. Thirty-seven independent studies consisted of 20 Asians, 10 Europeans, 3 Americans, 3 Africans and 1 mixed population. All of the studies used peripheral blood samples for DNA extraction, and the polymerase chain reaction method, TaqMan, or DNA sequencing methods were utilized for genotyping. Each single case was definitively diagnosed by qualified medical institutions. Some of papers divided male infertility into two types: azoospermia and oligoasthenoteratozoospermia (OAT). Also, healthy controls were defined as fertile men and were population-based in most works. In addition, the distribution of genotypes in all of the controls was consistent with HWE, except for nine case-control studies. The main characteristics for all eligible studies are listed in Supplemental Table 1.

### MTHFR C677T (rs1801133) and A1298C (rs1801131)

**Figure 3:**
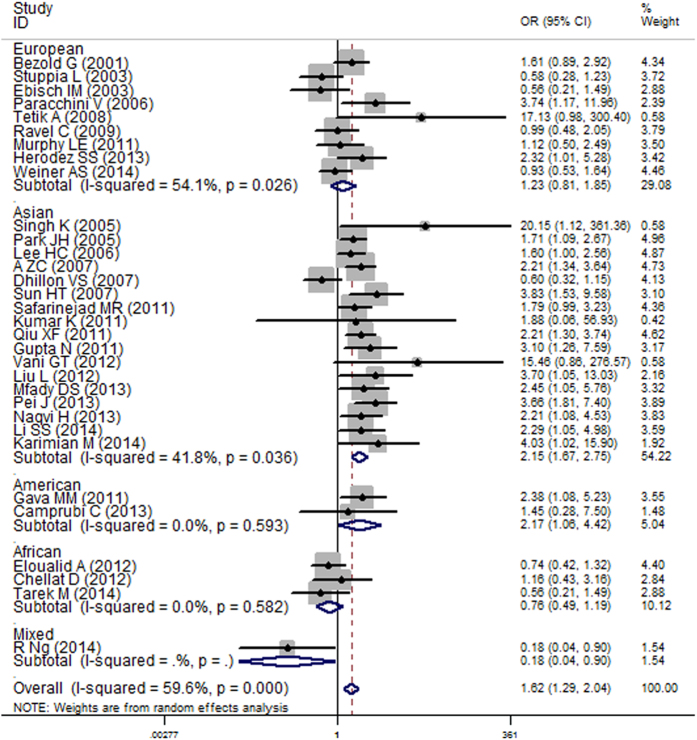
Forest plot of the association between the MTHFR C677T mutation and male infertility stratified by ethnicity (homozygote model).

Table 1 lists the main results of the meta-analysis of the associations between the C677T mutation and male infertility risk. Overall, a significantly increased risk of male infertility was associated with the C677T mutation in all of the genetic models. In the subgroup analysis by ethnicity, the TT genotype was a risk factor in Asians for all genetic models (Fig. 3, homozygote model, O = 1.62, 95% CI = 1.29–2.04, P < 0.001) and in Americans for the homozygote comparison but a protective factor in Africans in the dominant model and allele comparison. After multiple testing adjustment, the associations between the American (p = 0.033, FDR = 0.062) and African (p = 0.036, FDR = 0.090) populations and male infertility tend to be insignificant. No obvious association was observed in European populations. Furthermore, in the stratified analysis by sample size, we found a markedly increased risk in both the large and small sample size groups for all of the genetic models except the heterozygote comparison. In terms of azoospermia, significant associations were observed in all of the genetic models, especially in Asians and the large sample size subgroup. Similar results were observed in the OAT patients.

It was worth noting that there was significant heterogeneity in all of the genetic models. The subgroup analysis failed to reduce the heterogeneity. We then conducted Galbraith plot and meta-regression analysis to probe the source of heterogeneity. We did not find any explicit source of heterogeneity at last. Unacceptable heterogeneity was also observed when we investigated the associations between azoospermia and OAT with the C677T mutation. Through stratified analysis, the heterogeneity of the subgroup was decreased. The results of the meta-regression analysis suggested that ethnicity contributed to substantially altered heterogeneity, which was in accordance with the outcomes of the stratified analysis.

For the MTHFR A1298C mutation, five types of genetic models did not produce any significant association among all of the eligible studies (Supplemental Figure 1A, recessive model, OR = 1.11, 95% CI = 0.87–1.41, FDR = 0.792), and no positive result was obtained after the subgroup analyses. Moreover, we did not find any meaningful associations between azoospermia and OAT with the A1298C mutation. As shown in Supplemental Table 2, the heterogeneity across all of the studies was not prominent after the subgroup analyses.

### MTR A2756G (rs1805087) and MTRR A66G (rs1801394)

**Figure 4:**
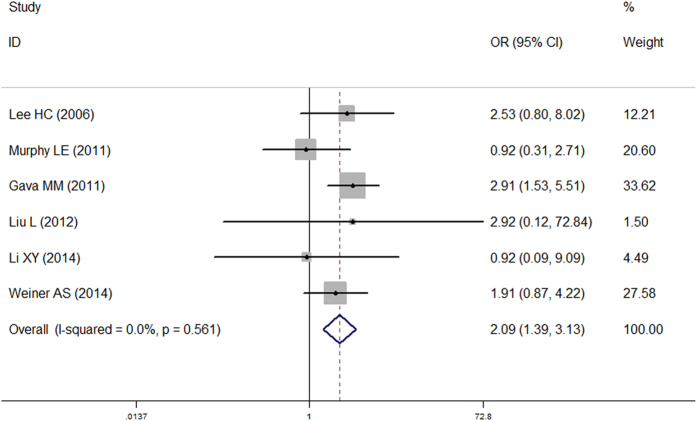
Forest plot of the association between the MTR A2756G mutation and male infertility (recessive model).

As shown in Table 2, the overall OR with its 95% CI exhibited statistically significant association between the MTR A2756G mutation and an increased risk of male infertility for the homozygous genetic model (OR = 2.05, 95% CI = 1.36–3.09, FDR = 0.003), the recessive genetic model (Fig. 4, OR = 2.09, 95% CI = 1.39–3.13, FDR < 0.001) and allele contrast (OR = 1.28, 95% CI = 1.09–1.50, FDR = 0.003). Because a departure from HWE may be a potential cause of inconsistent results, we excluded the studies that were inconsistent with HWE; however, the results did not change. Interestingly, when restricting the analysis to ethnicity, the association between the MTR A2756G mutation and male infertility became insignificant. In addition, the results indicated that individuals with the AA genotype were more susceptible to azoospermia than those carried GG genotype. But after removing the studies that did not conform to HWE, the positive associations were no longer observed. In the OAT subgroup, significant association was only observed in the homozygous genetic model; however, the association became meaningless after multiple testing adjustment (p = 0.043, FDR = 0.108). Heterogeneity analysis verified that the heterogeneity across researches was mild (recessive model: p = 0.561).

With regard to the MTRR A66G mutation, we did not detect any outstanding association between the A66G mutation and male infertility risk in the overall analyses for all of the genetic models (Supplemental Table 3). However, in the subgroup analysis by ethnicity, an increased risk was observed in Asians but not in Europeans in the homozygous genetic model (Supplemental Figure 1B, OR = 1.61, 95% CI = 1.04–2.50), the dominant genetic model (OR = 1.37, 95% CI = 1.02–1.84) and allele contrast (OR = 1.20, 95% CI = 1.00–1.43); however, the positive results were no longer obtained after adjusting for multiple comparisons. No significant risks were observed among the azoospermia and OAT types. Furthermore, the heterogeneity analysis stated that all of studies had favorable homogeneity (homozygous model: p = 0.450).

### Association between MTHFR haplotype and male infertility

In this meta-analysis, we also examined the relationship between the MTHFR haplotype and male infertility. The main characteristics for all of the eligible studies and the numbers of individuals carried different MTHFR haplotype are listed in Table 3a. Crucial results were summarized in Table 3b. Striking differences were observed in CC vs. CA (OR = 0.77, 95% CI = 0.65–0.91), TC vs. CA (OR = 2.09, 95% CI = 1.11–3.93), CC vs. TA (OR = 0.80, 95% CI = 0.66–0.97), CC vs. TC (OR = 0.41, 95% CI = 0.31–0.55) and TA vs. TC (OR = 0.54, 95% CI = 0.42–0.71). When we restricted the analysis to Asians, we obtained similar results for CC vs. CA, CC vs. TC and TA vs. TC.

### Sensitivity analysis

In the sensitivity analysis (Supplemental Figure 2), the influence of each study on the pooled OR was assessed by repeating the meta-analysis while omitting each study, one at a time. This procedure certified that our results were reliable and robust. In the meanwhile, we conducted leave-one-out sensitivity analyses in the azoospermia and OAT groups. The analysis outcomes further confirmed the stability of the results of our meta-analysis.

### Publication bias

**Figure 5:**
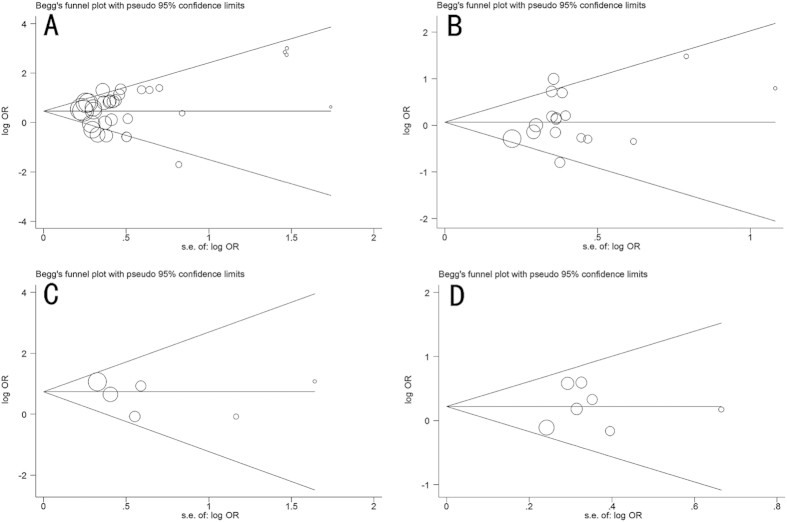
Begg’s funnel plot for publication bias test. (**A**) MTHFR C677T mutation (**B**) MTHFR A1298C mutation (**C**) MTR A2756G mutation (**D**) MTRR A66G mutation. Each circle represents a separate study. The area of each circle represents the contribution of the study to the pooled OR.

Begg’s funnel plot and Egger’s test were performed to evaluate the publication bias of the literatures. As shown in Fig. 5, the shapes of the funnel plots seemed symmetrical. Then, the Egger’s test was used to provide statistical evidence of funnel plot symmetry. The results still did not reveal any evidence of publication bias (C677T: Begg p = 0.140, Egger p = 0.304, Fig. 5A; A1298C: Begg p = 0.592, Egger p = 0.226, Fig. 5B; A2756G: Begg p = 0.707, Egger p = 0.438, Fig. 5C; A66G: Begg p = 1.000, Egger p = 0.829, Fig. 5D). Similarly, no publication bias was detected for the association of mutations in the folate-related enzyme genes with azoospermia and OAT.

### Trial sequential analysis

**Figure 6:**
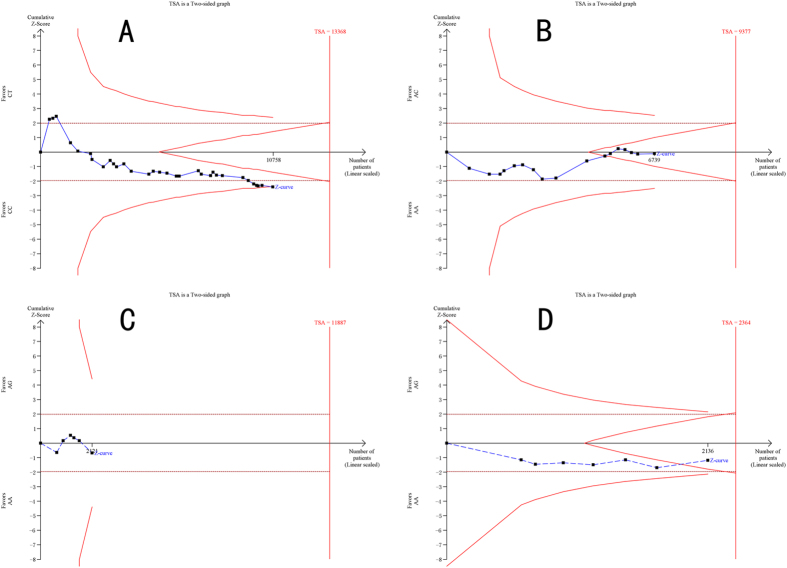
Trial sequential analyses of (**A**) the MTHFR C677T mutation; (**B**) the MTHFR A1298C mutation; (**C**) the MTR A2756G mutation; and (**D**) the MTRR A66G mutation and male infertility risk in the heterozygote model. The heterogeneity corrected optimal information size was based on a relative risk reduction of 10%, an alpha of 5% and a beta of 5%. The blue dash line represents the cumulative Z-score of the meta-analysis. The red straight lines represent the conventional p = 0.05 statistical boundaries. The inward sloping red lines represent the truncated trial sequential monitoring boundaries.

For the MTHFR C677T mutation, we finally calculated the required information size to 13368 patients. Although the number of patients included in our study did not exceed the required information size, the blue cumulative Z curve crossed the red trial sequential monitoring boundary, which confirmed our positive results (Fig. 6A). As for the MTHFR A1298C and MTRR A66G mutations, the number of patients did not reach optimal information size; however, the blue cumulative Z curve crossed the red inner wedge boundary for futility showing sufficient evidence that there was no difference between individuals with a wild-type genotype and individuals carrying a mutant allele (Fig. 6B,C). With respect to the MTR A2756G mutation, actually accrued number of participants only accounted for approximately 20% of the required information size. The blue cumulative Z curve did not cross the trial sequential monitoring boundary (Fig. 6D).

## Discussion

Spermatogenesis is a complex forming process of haploid spermatozoa from mitotic and meiotic divisions of germ cells, which represents one of the most hopeful areas of research in the genetics of male infertility^63^. The whole course of sperm formation involves distinct cellular, genetic, and chromatin changes^64^. The defects in spermatogenesis range from azoospermia, with an absence of mature germ cells, to oligozoospermia, which is the formation of a deficient number of sperm^51^,^65^,^66^. Nevertheless, the pathogenesis of male infertility is still not fully understood. Previous studies have shown that the folate metabolic pathway plays an important role in spermatogenesis^67^. It is widely shared that mutations in the genes encoding key enzymes involved in folate metabolism are potential risk factors for male infertility^18^. Of which, MTHFR C677T, MTHFR A1298C, MTR A2756G and MTRR A66G mutations are most studied. Meta-analysis is a favorable tool to increase the statistical power of all eligible studies and to summarize the existing evidence^68^. We discovered that previous meta-analyses on MTHFR C677T and A1298C mutations got opposite conclusions, and none of the meta-analyses included comprehensive eligible studies. The lack of eligible studies might cause a deviation in the final result and might draw false positive conclusions^69^. Moreover, no meta-analysis focused on the MTHFR haplotype, MTR A2756G and MTRR A66G mutations. To fill these gaps, we performed a comprehensive literature search and trial sequential analysis to pursue more precise results. Ultimately, we drew the conclusion that the MTHFR C677T mutation was a risk factor for male infertility in both azoospermia and OAT patients; however, ethnic differences could not be ignored. MTHFR A1298C mutation was not related to male infertility. Furthermore, the MTR A2756G and MTRR A66G mutations were potentially linked with a risk of male infertility. It was worth noting that the links turned out to be false positive when using TSA to confirm, and thus further exploration was required. Men carrying the MTHFR TC haplotype were most liable to suffer infertility, whereas those with the CC haplotype had the lowest hazard.

### The MTHFR C677T mutation

The MTHFR C677T mutation was first studied by Bezold *et al.* in relation to male fertility by comparing 255 patients seeking a fertility evaluation to 200 controls in 2001^33^. Bezold *et al.* advocated a possible role of this mutation in the pathogenesis of male infertility in German. In contrast, this conclusion was not shared by an Indian research group who demonstrated a lack of an association of the C677T mutation with male infertility^49^. Hence, the results seemed to be influenced by the different races. In our meta-analysis, we also found noteworthy discrepant outcomes among the different ethnicities, although the overall OR with its 95% CI was statistically significant. One factor that might contribute to the discrepancy was that participants from different ethnic groups had diverse cultural, environmental and genetic characteristics. Namely, the prevalence of the homozygous TT allele was likely to vary substantially among different racial populations. Botto and Yang reported that the frequency of TT genotype ranged from 1% or less among Blacks from Africa and the USA to 20% or more among Italians and US Hispanics^70^. Furthermore, other researchers noted that the C677T mutation was related to folate status^71^. Munoz-Moran E. and colleagues argued that the number of individuals with a mutated genotype doubled accompanying with the development of folic acid supplementation programs, which verified a possible genetic selection by changes in the diet and folate intake^72^. Comparatively speaking, Western European people have a better nutritional status, which may account for a higher frequency of the TT genotype. Thus, the phenomenon of gene-nutrient interaction can partly explain the discrepant allele distribution among different ethnicities^73^. On the other hand, we noted that the sample size and numbers of studies in American and African groups were not ample, which might result in mistaken conclusions^74^. Especially after multiple testing adjustment, the significant associations seemed to be false-positive. Even so, we could not completely deny the association between the MTHFR C677T mutation and the susceptibility of male infertility in American and African populations because of the lack of an enough sample size. Moreover, selection bias, different matching criteria and inaccurate genotyping methods might affect the accuracy of our results^21^. For example, according to the WHO classification system, male infertility can be diagnosed as OAT, azoospermia and the like; however, each type of dysspermia has its own characteristic and may differ from each other^75^,^76^. Therefore, the effects of genetic mutations on different types of male infertility may be distinct. If two case groups have similar sample size, but the proportions of different types of dysspermia differ greatly, two conclusions can be opposite. In brief, the negative association was likely to become false-positive or be over-estimated^77^.

### The MTHFR A1298C mutation and combined analysis

The A1298C mutation leads to a glutamine to alanine change at codon 429 and is detected in a regulatory region of the MTHFR enzyme. Eloualid *et al.* and Singh K *et al.* emphasized the relationship between the A1298C mutation and an increased hazard of male infertility in Moroccan and Indian populations, respectively^47^,^59^. Unfortunately, we failed to detect any sensible associations even in the subgroup analysis, and further TSA notarized our negative results. We speculated that a single case-control study with relatively small sample size and specific race might over-estimate the association. Apart from genetic aspect, the environmental factors might play an important role in male infertility^78^. Manfo FP *et al.* state that the environmental contaminants had serious impact on the male reproductive function^79^. If individuals who lived in the polluted environment were recruited in the case-control study, the conclusion might be influenced by confounders and was unreliable. Additionally, unhealthy lifestyles such as sedentariness and smoking, ejaculatory frequency and scrotal temperature were important factors in the development of male infertility^80^. The influence of these confounding factors was easily mistaken for the effect of genetic mutations. Interestingly, in the haplotype analysis, we probed that individuals carrying the TC haplotype were more likely than those with TA to suffer from male infertility. It was suggested the C allele could be a risk factor in the occurrence of male infertility. The C677T and C1298A mutations show an intermediate concentration of linkage disequilibrium LD (D’ = 0.586, r^2^ = 0.056)^60^. As is well known, the combined effects of SNPs and SNP-SNP interaction cannot be neglected. More studies with a larger sample size are needed to identify the role of the MTHFR haplotype in the risk of developing male infertility.

### The MTR and MTRR mutations

MTR and MTRR, two important regulatory enzymes in the homocysteine metabolic pathway, map to chromosomes 1q43 and 5p15.31, respectively^81^. MTR plays a critical role in methyl group metabolism because it catalyzes the remethylation of homocysteine (Hcy) to methionine. The function of MTRR is to maintain the active state of MTR through the reductive methylation of cob(II)alamin. Both MTRR and MTR maintain a balance of Hcy in the body^17^,^67^. In our meta-analysis, we found that MTR A2756G was associated with an increased risk of azoospermia and OAT.; however, when we removed the studies that did not conform to HWE, the overall ORs with their 95% CIs became meaningless. Meanwhile, the significant relation between A2756G and OAT was no longer observed after using BH method. How is it possible to reconcile the odd scenario? We hypothesized that the small sample size might cause these false-positive associations. Furthermore, trial sequential analysis demonstrated that our meta-analysis neither included adequate participants nor obtained exact conclusions in advance, thus conforming our hypothesis. Apart from this reason, the study population compositions, the techniques used and the combined effects of SNPs possibly contributed to the false-positive results. In terms of the MTRR A66G mutation, it was first reported by Lee HC *et al.* and the authors stated a significant association between this mutation and male infertility in an Asian population^18^. On the contrary, a similar relationship was not observed in non-Asian populations^8^,^15^. We did not find any meaningful association in both Asian and non-Asian populations in our study. Although TSA affirmed our negative results, we advised that more large-scale experiments were still wanted to support this conclusion.

### The mechanism of associations between mutations in folate-related enzyme genes and male infertility

The homocysteine/methionine cycle is one of the most important pathways of Hcy in most mammalian cells^82^. This cycle’s turning accurately relies on the normal functioning of another cycle, the folate cycle. Folate deficiency and the subsequent accumulation of Hcy are deemed to be relevant to impaired sperm parameters and male infertility^11^,^83^. A classic MTHFR knockout mouse model study also supports these viewpoints^84^. Previous works have highlighted that mutations in folate-related enzyme genes are involved in the occurrence of male infertility via leading to excessive reactive oxygen species (ROS) production and aberrant methylation, which result in abnormal DNA replication, repair, transcription, and other issues^67^. As far as we know, The TT genotype of the C677T mutation may cause only 30% enzymatic activity compared to the wild-type genotype^33^. We performed a secondary structure of the MTHFR mRNA sequence prediction ( http://rna.tbi.univie.ac.at/ cgi-bin/.RNAfold.cgi)^85^ and found that the minimum free energy changed when substituting the C allele with the T allele, suggesting that the C677T mutation might impact the stability of the RNA. Our meta-analysis confirmed an association between the C677T mutation and risk of male infertility. Moreover, A1298C, another mutation of MTHFR, was reported to reduce the enzymatic activity as well, but to a lesser extent than C677T^86^. However, this mutation was not related to male infertility in our meta-analysis. We speculated that the effect of A1298C on decreased enzymatic activity was too subtle to further influence homocysteine metabolism. The haplotype analysis showed a SNP-SNP interaction was supposed to receive due attention. A2756G and A66G, two key mutations in the folate cycle, might also affect the functions of MTR and MTRR, respectively, which further result in reduced enzymatic activity; but the lack of a sufficient sample size deterred us from exploring a detailed mechanism of these two mutations in this analysis.

### Limitations of this study

Despite the overall robust statistical evidence generated through this analysis, some limitations should be addressed. First, identifying the source of heterogeneity was one of the most important goals of this meta-analysis. We only observed significant heterogeneity among all of the genetic models for the C667T mutation. Regretfully, despite great efforts, we failed to identify the source of heterogeneity. Incomplete data and redundant confounding factors might play a role to some degree. Next, our results were based on unadjusted estimates, while a more precise analysis should be conducted if all individual raw data were available, which would allow for the adjustment by other covariates, including folic acid and vitamin intake, drinking status, cigarette consumption, and other lifestyle factors. Third, male infertility is a broad concept that includes many types of disease, such as azoospermia, oligozoospermia, and teratozoospermia, among others. Additionally, male infertility is a multi-factorial disease that results from complex interactions among many genetic and environmental factors. Therefore, a more detailed subgroup analysis and combined effects analysis of the different SNPs are required. Last but not the least, an inadequate sample size may cause false-positive results. Although TSA is applied in our analysis, more studies involving A2756G, A66G, and American or African populations should be included.

### Conclusion, future and recommendations

In conclusion, our meta-analysis with trial sequential analysis demonstrated the important role of genetic mutations in folate-related enzyme genes in male infertility. The identification of male infertility susceptible variants can provide new insight into its etiology. Well-established genetic markers surely would contribute to the early screening and prediction of male infertility. Although, many questions remain unanswered, we believe that genetic mutations in the folate-related enzyme genes are a promising avenue in the research on male infertility. Hence, we recommend the following: first, male infertility contains various diseases, each with a distinct pathogenesis. We should divide patients into corresponding groups based on the different types of male infertility and perform more case-control studies or comprehensive updated meta-analyses to probe the underlying mechanisms. Second, we should attempt to prevent false-positive and negative results by conducting the studies using a large sample with stratification by age, work environment, food habit, lifestyle and ethnicity. Lastly, as genetic background of male infertility is a complicated issue, the combined effects of different SNPs and congenital chromosomal abnormalities cannot be overlooked.

## Additional Information

**How to cite this article**: Liu, K. *et al.* Role of genetic mutations in folate-related enzyme genes on Male Infertility. *Sci. Rep.* **5**, 15548; doi: 10.1038/srep15548 (2015).

## Supplementary Material

Supplementary Information

## Figures and Tables

**Figure 1:**
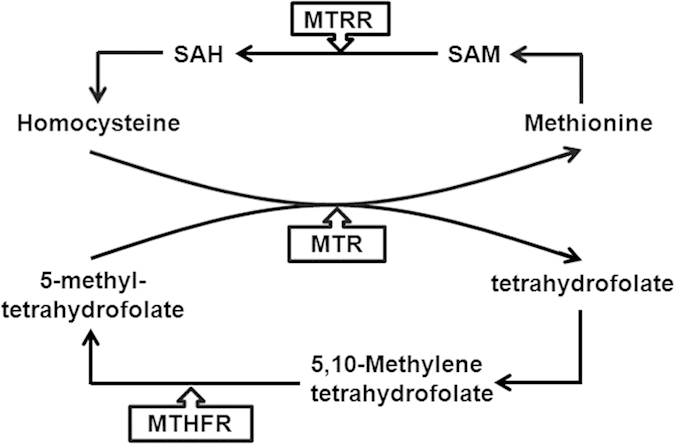
The important roles of three enzymes (MTHFR, MTR and MTRR) in folate metabolism. MTHFR catalyzes the reduction of methylenetetrahydrofolate (5,10-methyl THF) to methyltetrahydrofolate (5-methyl THF), which then donates a methyl group. MTR can catalyze the transfer of the methyl group from 5-methyl THF to homocysteine, which generates methionine and THF. MTRR is responsible to catalyzes the reductive methylation of MTR, which and maintains MTR in an active state during the folate cycle.

**Table 1:** Main results for the MTHFR C677T mutation in the meta-analysis.

MTHFR C677T(rs18013133)	N^a^	TT vs CC			CT vs CC			CT/TT vs CC			TT vs CT/CC			T vs C		
Variables		OR(95%CI)	P^b^	FDR^g^	OR(95%CI)	P^b^	FDR^g^	OR(95%CI)	P^b^	FDR^g^	OR(95%CI)	P^b^	FDR^g^	OR(95%CI)	P^b^	FDR^g^
**Total**	32	**1.62(1.29-2.04)**	0.000	0.000	**1.17(1.03-1.33)**	0.000	0.016	**1.26(1.10-1.45)**	0.000	0.001	**1.47(1.23-1.77)**	0.002	0.000	**1.25(1.12-1.40)**	0.000	0.000
**Consistent to HWE**	29	**1.70(1.35-2.14)**	0.000	0.000	**1.15(1.01-1.30)**	0.001	0.068	**1.26(1.09-1.45)**	0.000	0.002	**1.53(1.29-1.83)**	0.015	0.000	**1.27(1.14-1.42)**	0.000	0.000
**Ethnicity**																
European	9	1.23(0.81-1.85)	0.026	0.328	1.03(0.80-1.32)	0.046	0.978	1.08(0.84-1.40)	0.020	0.660	1.18(0.84-1.66)	0.085	0.411	1.10(0.91-1.34)	0.010	0.364
Asian	17	**2.15(1.67-2.75)**	0.036	0.000	**1.28(1.13-1.46)**	0.153	0.000	**1.44(1.24-1.66)**	0.019	0.000	**1.79(1.48-2.16)**	0.193	0.000	**1.42(1.27-1.60)**	0.005	0.000
American	2	2.17(1.06-4.42)	0.593	0.062	1.13(0.24-5.23)	0.004	0.978	1.27(0.33-4.78)	0.009	0.729	1.87(0.94-3.73)	0.830	0.125	1.42(0.67-3.00)	0.036	0.364
African	3	0.76(0.49-1.19)	0.582	0.296	0.78(0.61-1.01)	0.532	0.145	0.77(0.61-0.98)	0.555	0.090	0.84(0.54-1.29)	0.584	0.422	0.82(0.68-1.00)^c^	0.507	0.075
**Sample Size**																
≧500	7	**1.55(1.11-2.18)**	0.023	0.010	1.19(0.97-1.46)	0.014	0.093	**1.26(1.00-1.57)**^d^	0.002	0.045	**1.40(1.07-1.84)**	0.095	0.015	**1.23(1.03-1.47)**	0.001	0.024
<500	25	**1.66(1.22-2.25)**	0.000	0.002	1.16(0.98-1.37)	0.001	0.093	**1.26(1.05-1.51)**	0.000	0.024	**1.50(1.18-1.91)**	0.004	0.002	**1.26(1.09-1.46)**	0.000	0.004
**Subgroup**																
**Azoospermia**	14	**1.64(1.12-2.42)**	0.002	0.016	**1.21(1.04-1.41)**	0.298	0.016	**1.31(1.06-1.61)**	0.015	0.016	**1.49(1.07-2.06)**	0.011	0.017	**1.27(1.05-1.54)**	0.000	0.016
Consistent to HWE	11	**1.83(1.25-2.67)**	0.018	0.004	**1.21(1.03-1.42)**	0.209	0.044	**1.33(1.05-1.67)**	0.018	0.032	**1.63(1.21-2.20)**	0.092	0.002	**1.32(1.09-1.59)**	0.002	0.008
European	3	0.99(0.35-2.82)	0.139	0.992	1.09(0.77-1.53)	0.647	0.635	1.07(0.76-1.51)	0.344	0.829	0.94(0.36-2.44)	0.166	0.905	1.08(0.73-1.59)	0.122	0.716
Asian	6	**2.56(1.93-3.40)**	0.820	0.000	**1.46(1.19-1.80)**	0.824	0.000	**1.69(1.39-2.06)**	0.825	0.000	**2.04(1.59-2.61)**	0.689	0.000	**1.59(1.39-1.82)**	0.807	0.000
African	3	0.81(0.47-1.38)	0.558	0.540	0.79(0.57-1.09)	0.700	0.248	0.79(0.58-1.06)	0.754	0.198	0.89(0.53-1.49)	0.492	0.811	0.84(0.66-1.07)	0.625	0.252
≧300	8	**1.99(1.34-2.95)**	0.032	0.002	**1.24(1.04-1.48)**	0.119	0.028	**1.40(1.07-1.83)**	0.013	0.028	**1.72(1.27-2.35)**	0.116	0.002	**1.37(1.11-1.69)**	0.003	0.008
**OAT**^f^	16	**1.52(1.12-2.06)**	0.008	0.018	1.17(0.96-1.44)	0.001	0.127	1.25(1.01-1.55)	0.000	0.053	**1.43(1.13-1.82)**	0.098	0.015	1.24(1.05-1.47)	0.000	0.058
Consistent to HWE	14	**1.56(1.15-2.11)**	0.017	0.008	1.11(0.92-1.34)	0.016	0.542	1.21(0.99-1.48)	0.001	0.118	**1.47(1.17-1.86)**	0.153	0.002	**1.24(1.06-1.45)**	0.000	0.016
European	3	1.49(0.63-3.50)	0.141	0.369	0.98(0.69-1.38)	0.345	0.906	1.13(0.73-1.75)	0.163	0.592	1.47(0.71-3.04)	0.196	0.503	1.44(0.82-1.86)	0.071	0.304
Asian	9	**1.78(1.25-2.52)**	0.034	0.005	1.20(0.96-1.51)	0.023	0.275	**1.32(1.05-1.67)**	0.006	0.048	**1.61(1.24-2.08)**	0.196	0.000	**1.32(1.11-1.58)**	0.005	0.005
African	2	0.74(0.38-1.43)	0.666	0.369	0.78(0.54-1.14)	0.305	0.337	0.77(0.55-1.08)	0.315	0.158	0.81(0.42-1.53)	0.875	0.511	0.82(0.63-1.07)	0.384	0.171
≧300	9	1.39(1.00-1.93)^e^	0.051	0.084	1.16(0.88-1.51)	0.000	0.287	1.20(0.93-1.57)	0.000	0.168	1.33(1.03-1.71)	0.240	0.055	1.20(0.98-1.46)	0.000	0.113

^a^Number of studies.

^b^The value of heterogeneity test.

^c^The exact value is 0.823(0.681-0.996).

^d^The exact value is 1.257(1.005-1.571).

^e^The exact value is 1.390(1.001-1.929).

^f^Including OAT, severe OAT, oligozoospermia, and teratozoospermia.

^g^p value in multiple testing (Benjamini-Hochberg methods).

**Table 2:** Main results for the MTR A2756G mutation in the meta-analysis.

MTR A2756G(rs1805087)	N^a^	GG vs AA			AG vs AA			AG/GG vs AA			GG vs AG/AA			G vs A		
Variables		OR(95%CI)	P^b^	FDR^d^	OR(95%CI)	P^b^	FDR^d^	OR(95%CI)	P^b^	FDR^d^	OR(95%CI)	P^b^	FDR^d^	OR(95%CI)	P^b^	FDR^d^
**Total**	6	**2.05(1.36-3.09)**	0.615	0.003	1.07(0.88-1.31)	0.408	0.483	1.20(0.99-1.44)	0.666	0.071	**2.09(1.39-3.13)**	0.561	0.000	**1.28(1.09-1.50)**	0.353	0.003
**Consistent to HWE**	5	**1.74(1.03-2.22)**	0.627	0.040	1.14(0.92-1.41)	0.665	0.217	1.20(0.98-1.47)	0.522	0.166	1.67(0.99-2.83)	0.708	0.055	**1.22(1.02-1.45)**	0.433	0.030
**Ethnicity**																
Asian	3	2.23(0.83-5.96)	0.736	0.165	1.17(0.85-1.60)	0.945	0.437	1.24(0.91-1.67)	0.961	0.410	2.17(0.81-5.78)	0.728	0.185	1.27(0.97-1.68)	0.929	0.128
European	2	1.55(0.82-2.92)	0.200	0.174	1.12(0.84-1.49)	0.135	0.437	1.17(0.89-1.53)	0.080	0.410	1.49(0.79-2.79)	0.285	0.215	1.18(0.93-1.48)	0.062	0.166
**Subgroup**																
**Azoospermia**	4	**2.25(1.36-3.73)**	0.744	0.005	1.06(0.80-1.41)	0.622	0.700	1.23(0.95-1.60)	0.972	0.153	**2.31(1.41-3.79)**	0.592	0.001	**1.35(1.09-1.68)**	0.602	0.007
Consistent to HWE	3	1.83(0.95-3.52)	0.878	0.069	1.13(0.83-1.54)	0.813	0.426	1.21(0.91-1.62)	0.919	0.379	1.79(0.94-3.42)	0.853	0.079	1.25(0.98-2.60)	0.988	0.076
**OAT**^c^	5	1.70(1.02-2.83)	0.146	0.108	0.96(0.73-1.26)	0.723	0.780	1.06(0.83-1.37)	0.569	0.780	1.77(1.07-2.93)	0.146	0.108	1.16(0.93-1.43)	0.128	0.308
Consistent to HWE	4	1.29(0.63-2.63)	0.095	0.481	1.03(0.76-1.38)	0.804	0.862	1.05(0.79-1.39)	0.409	0.751	1.28(0.63-2.59)	0.113	0.492	1.07(0.83-1.37)	0.125	0.606

^a^Number of studies.

^b^The value of heterogeneity test.

^c^Including OAT, severe OAT, oligozoospermia, and teratozoospermia.

^d^p value in multiple testing (Benjamini-Hochberg methods).

**Table 3:** **(A)** Main characteristics of the studies of the MTHFR haplotype included in the meta-analysis; **(B)** Main results for the MTHFR haplotype in the meta-analysis.

**A**													
Name	Year	Country	Ethnicity	Case	Con	CA1	CC1	TA1	TC1	CA0	CC0	TA0	TC0
Dhillon VS	2007	India	Asian	179	200	182	57	78	41	164	76	126	34
Safarinejad MR	2011	Iran	Asian	164	328	165	31	55	77	336	100	103	117
Eloualid A	2012	Morocco	African	257	690	305	89	92	28	801	319	252	8
Herodez SS	2013	Slovenia	European	100	111	69	39	53	37	95	49	51	27
Mfady DS	2013	Jordan	Asian	150	150	106	90	96	8	112	103	81	4
**B**													
**MTHFR haplotype**		**CC vs CA**		**TA vs CA**		**TC vs CA**		**CC vs TA**		**CC vs TC**		**TA vs TC**	
**Variables**	**N**^a^	**OR(95%CI)**	**P**^b^	**OR(95%CI)**	**P**^b^	**OR(95%CI)**	**P**^b^	**OR(95%CI)**	**P**^b^	**OR(95%CI)**	**P**^b^	**OR(95%CI)**	**P**^b^
Total	5	**0.77(0.65-0.91)**	0.420	0.99(0.73-1.34)	0.008	**2.09(1.11-3.93)**	0.000	**0.80(0.66-0.97)**	0.281	**0.41(0.31-0.55)**	0.001	**0.54(0.42-0.71)**	0.001
Asian	3	**0.74(0.59-0.94)**	0.380	0.91(0.55-1.50)	0.005	1.29(0.98-1.70)	0.572	0.82(0.63-1.05)	0.085	**0.52(0.36-0.74)**	0.738	**0.67(0.48-0.93)**	0.421

^a^Number of studies.

^b^The value of heterogeneity test.
