# Supplementary material for: Role of genetic mutations in folate-related enzyme genes on Male Infertility
Source: Sci Rep. 2015 Nov 9;5:15548. doi: 10.1038/srep15548 (PMC4637885; doi:10.1038/srep15548)
Supplement: Supplementary Information [file srep15548-s1.pdf]

Role of genetic mutations in folate-related enzyme genes on Male Infertility

Kang Liu[☯], Ruizhe Zhao[☯], Min Shen[☯], Jiaxin Ye, Xiao Li, Yuan Huang, Lixin Hua, Zengjun Wang, Jie Li*

Department of Urology, The First Affiliated Hospital of Nanjing Medical University, Nanjing, China.

☯ These authors contributed equally to this work.

* E-mail: lijie_urology@163.com.

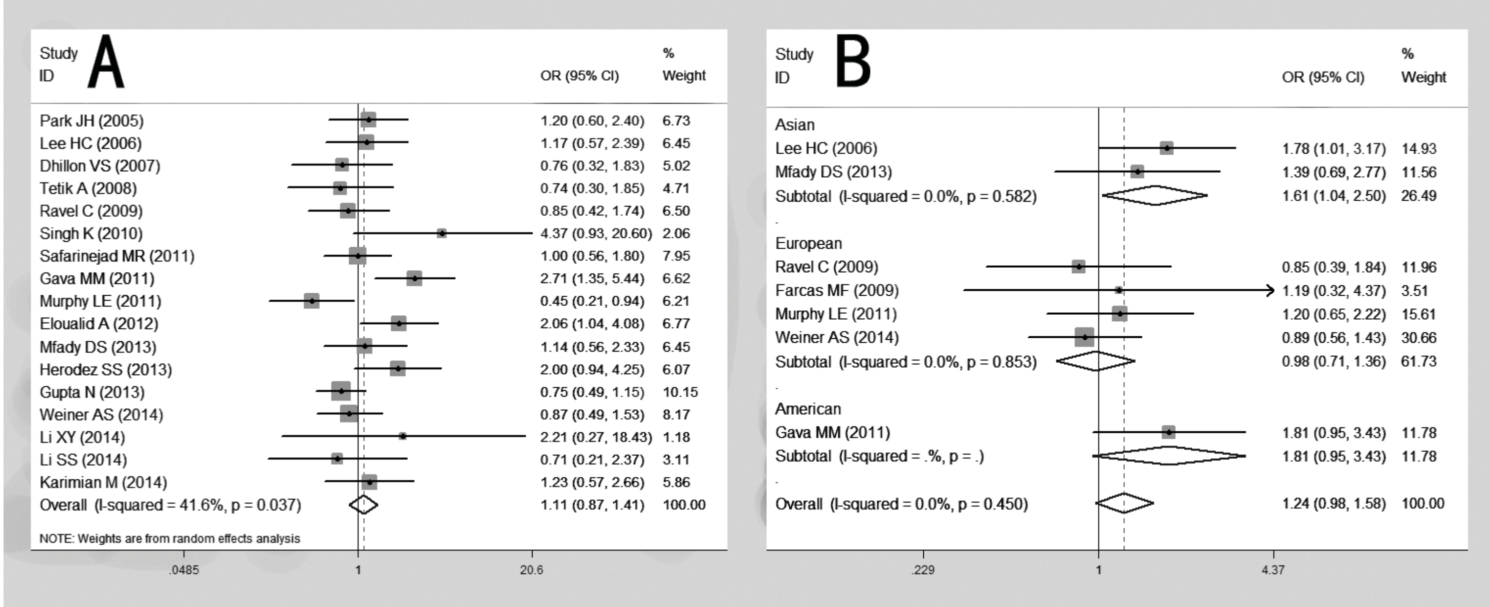

Supplemental Figure 1 (A) Forest plot of the association between the MTHFR A1298C mutation and male infertility (recessive model); **(B)** Forest plot of the association between the MTRR A66G mutation and male infertility stratified by ethnicity (homozygote model).

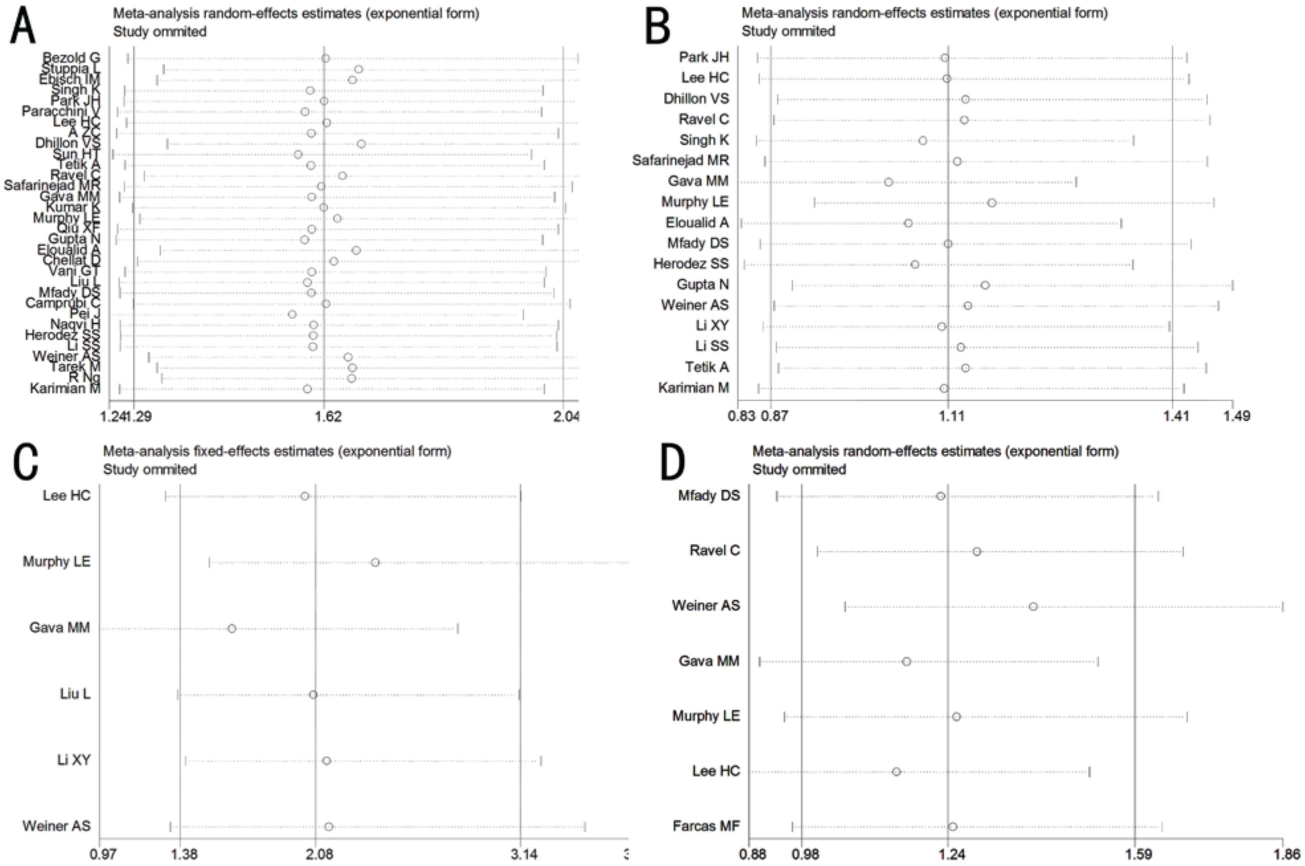

Supplemental Figure 2 (A) Sensitivity analysis of male infertility risk associated with the MTHFR C677T mutation under the homozygote model; **(B)** Sensitivity analysis of male infertility risk associated with the MTHFR A1298C mutation under the recessive model; **(C)** Sensitivity analysis of male infertility risk associated with the MTR A2756G mutation under the recessive model; **(D)** Sensitivity analysis of male infertility risk associated with the MTRR A66G mutation under the homozygote model.

Supplemental Table 1. Main characteristics of all studies included in the meta-analysis

Name	Year	Country	Ethnicity	Genotyping	Mutation	Case			Azoospermia			OAT			Control			HWE
						W/W	W/M	M/M	W/W	W/M	M/M	W/W	W/M	M/M	W/W	W/M	M/M	
Bezold G [33]	2001	Germany	European	PCR-RFLP	C677T	144	93	48	—	—	—	—	—	—	92	89	19	Y
Stuppia L [34]	2003	Italy	European	PCR-RFLP	C677T	37	37	19	—	—	—	—	—	—	33	43	29	Y
Ebisch IM [35]	2003	Netherlands	European	SSCP-PCR	C677T	42	28	7	—	—	—	—	—	—	50	48	15	Y
Singh K [36]	2005	India	Asian	PCR-RFLP	C677T	105	40	6	—	—	—	—	—	—	163	37	0	Y
Park JH [37]	2005	Korea	Asian	PCR-RFLP	C677T	105	205	63	—	—	—	—	—	—	145	200	51	Y
					A1298C	237	118	18	—	—	—	—	—	—	269	111	16	Y
Paracchini V [16]	2006	Italy	European	SSCP-PCR	C677T	11	32	16	—	—	—	—	—	—	18	21	7	Y
Lee HC [18]	2006	Korea	Asian	PCR-RFLP	C677T	115	181	64	44	100	30	71	81	34	118	166	41	Y
					A1298C	222	120	18	109	57	8	113	63	10	213	98	14	Y
					A2756G	270	79	11	124	41	9	146	38	2	255	66	4	Y
					A66G	64	250	46	38	121	15	26	129	31	72	224	29	N
A ZC [38]	2007	China	Asian	PCR-RFLP	C677T	130	160	65	83	97	48	47	63	17	128	95	29	Y
Dhillon VS [39]	2007	India	Asian	PCR-RFLP	C677T	81	77	21	—	—	—	81	77	21	70	100	30	Y
					A1298C	90	80	9	—	—	—	90	80	9	103	84	13	Y
Sun HT [40]	2007	China	Asian	PCR-RFLP	C677T	27	86	69	—	—	—	22	75	52	15	28	10	Y
					A1298C	45	41	14	21	23	6	24	18	8	19	22	9	Y
Tetik A [41]	2008	Turkey	European	PCR-RFLP	C677T	44	44	12	23	22	5	21	22	7	30	20	0	Y
Ravel C [17]	2009	French	European	PCR-RFLP	C677T	118	101	31	33	31	6	85	70	25	49	52	13	Y
					A1298C	131	94	25	34	28	7	97	66	18	54	46	13	Y
					A66G	27	132	80	8	39	19	19	93	61	12	57	42	Y
Farcas MF [11]	2009	Romania	European	PCR-RFLP	A66G	13	46	6	—	—	—	—	—	—	18	42	7	N
Singh K [59]	2010	India	Asian	PCR-RFLP	A1298C	66	76	9	66	76	9				64	74	2	N
Safarinejad MR [42]	2011	Iran	Asian	PCR-RFLP	C677T	58	80	26	—	—	—	58	80	26	144	148	36	Y
					A1298C	75	70	19	—	—	—	75	70	19	149	141	38	Y

Gava MM [43]	2011	Brazil	American	Taqman	C677T	81	60	15	27	15	7	54	45	8	167	53	13	N
					A1298C	71	62	23	26	14	9	45	48	14	130	89	14	Y
Gava MM [8]	2011	Brazil	American	Taqman	A2756G	78	23	32	31	9	15	47	14	17	109	47	17	N
					A66G	37	62	34	17	26	12	20	36	22	59	84	30	Y
Kumar K [44]	2011	India	Asian	PCR-RFLP	C677T	86	14	0.5	—	—	—	—	—	—	81	19	0	Y
Murphy LE [45]	2011	Sweden	European	Allele-specific PCR	C677T	73	63	13	—	—	—	—	—	—	94	73	15	Y
					A1298C	58	77	11	—	—	—	—	—	—	87	62	27	N
					A2756G	100	41	6	—	—	—	—	—	—	116	57	8	Y
					A66G	50	68	32	—	—	—	—	—	—	60	88	32	Y
Qiu XF [46]	2011	China	Asian	PCR-RFLP	C677T	75	112	84	42	66	50	33	46	34	63	85	32	Y
Gupta N [14]	2011	India	Asian	Sequencing	C677T	378	116	28	49	15	4	329	101	24	251	58	6	Y
Eloualid A [47]	2012	Morocco	African	PCR-RFLP	C677T	152	88	17	65	37	8	87	51	9	351	286	53	Y
					A1298C	205	122	17	67	39	4	138	83	13	370	303	17	N
Chellat D [48]	2012	Algeria	African	PCR-RFLP	C677T	31	33	10	20	19	7	11	14	3	36	38	10	Y
Vani GT [49]	2012	India	Asian	PCR-RFLP	C677T	158	42	6	—	—	—	—	—	—	188	42	0	Y
Liu L [50]	2012	China	Asian	PCR-RFLP	C677T	27	38	10	—	—	—	27	38	10	40	28	4	Y
					A2756G	60	14	1	—	—	—	60	14	1	61	11	0	Y
Mfady DS [51]	2013	Jordan	Asian	PCR-RFLP	C677T	67	63	20	—	—	—	—	—	—	74	67	9	Y
					A1298C	71	61	18	—	—	—	—	—	—	59	75	16	Y
					A66G	48	78	24	—	—	—	—	—	—	61	67	22	Y
Camprubi C [52]	2013	USA	American	PCR-RFLP	C677T	47	43	17	—	—	—	—	—	—	8	15	2	Y
Pei J [53]	2013	China	Asian	PCR-RFLP	C677T	39	138	113	39	138	113	—	—	—	24	47	19	Y
Naqvi H [54]	2013	India	Asian	PCR-RFLP	C677T	447	154	36	34	11	4	413	143	32	275	79	10	Y
Herodez SS [55]	2013	Slovenia	European	Allele-specific PCR	C677T	29	51	20	—	—	—	—	—	—	47	50	14	Y
					A1298C	44	35	21	—	—	—	—	—	—	48	50	13	Y
Gupta N [60]	2013	India	Asian	Sequencing	A1298C	165	320	126	13	33	8	73	141	61	27	74	35	Y
Li SS [56]	2014	China	Asian	PCR-RFLP	C677T	14	36	32	—	—	—	—	—	—	36	61	36	Y

					A1298C	49	29	4	—	—	—	—	—	—	88	36	9	Y
Weiner AS [15]	2014	Russia	European	Taqman	C677T	129	116	26	49	41	8	40	31	11	153	115	33	Y
					A1298C	126	125	23	37	54	8	42	32	9	142	142	30	Y
					A2756G	157	98	18	61	30	7	48	27	8	184	87	10	Y
					A66G	54	136	82	19	49	31	17	37	28	57	170	97	Y
Hussein TM [57]	2014	Egypt	African	PCR-RFLP	C677T	60	29	7	60	29	7	—	—	—	62	32	13	N
R Ng [58]	2014	Canada	Mixed	PCR-RFLP	C677T	22	14	3	10	10	2	12	4	1	8	5	6	N
Li XY [61]	2014	China	Asian	Taqman	A1298C	101	54	7	66	31	3	35	23	4	34	15	1	Y
					A2756G	124	35	3	74	24	2	50	11	1	40	9	1	Y
Karimian M [62]	2014	Iran	Asian	PCR-RFLP	C677T	51	59	8	—	—	—	—	—	—	77	52	3	Y
					A1298C	59	44	15	—	—	—	—	—	—	70	48	14	Y

Supplemental Table 2. Main results for the MTHFR A1298C mutation in the meta-analysis

MTHFR A1298C(rs18013131)		CC vs AA			AC vs AA			AC/CC vs AA			CC vs AC/AA			C vs A		
**Variables**	**N[a]**	**OR(95%CI)**	**FDR[d]**	**P[b]**	**OR(95%CI)**	**FDR[d]**	**P[b]**	**OR(95%CI)**	**FDR[d]**	**P[b]**	**OR(95%CI)**	**FDR[d]**	**P[b]**	**OR(95%CI)**	**FDR[d]**	**P[b]**
**Total**	17	1.09(0.86-1.39)	0.792	0.069	1.01(0.89-1.15)	0.896	0.094	1.02(0.92-1.12)	0.896	0.179	1.11(0.87-1.41)	0.792	0.037	1.06(0.96-1.17)	0.792	0.057
**Consistent to HWE**	14	1.05(0.83-1.34)	0.685	0.171	1.02(0.90-1.15)	0.789	0.559	1.04(0.93-1.16)	0.643	0.359	1.07(0.87-1.32)	0.582	0.282	1.08(0.96-1.22)	0.406	0.035
**Ethnicity**																
European	5	0.87(0.62-1.23)	0.546	0.389	1.02(0.74-1.41)	0.908	0.082	1.00(0.82-1.22)	0.975	0.355	0.87(0.55-1.38)	0.741	0.095	1.00(0.86-1.16)	0.970	0.773
Asian	10	0.99(0.76-1.27)	0.912	0.430	1.05(0.91-1.20)	0.701	0.488	1.05(0.92-1.19)	0.637	0.445	1.00(0.79-1.26)	0.982	0.605	1.08(0.94-1.23)	0.507	0.085
**Sample Size**																
≧500	5	1.04(0.70-1.53)	0.861	0.119	0.95(0.76-1.19)	0.653	0.057	0.96(0.84-1.11)	0.581	0.067	1.07(0.76-1.51)	0.704	0.155	0.98(0.85-1.13)	0.800	0.145
< 500	12	1.13(0.82-1.56)	0.861	0.098	1.05(0.90-1.24)	0.653	0.256	1.07(0.93-1.23)	0.581	0.356	1.12(0.80-1.57)	0.704	0.039	1.12(0.99-1.28)	0.156	0.137
**Subgroup**																
**Azoospermia**	9	1.16(0.82-1.64)	0.950	0.155	0.99(0.83-1.19)	0.950	0.666	1.02(0.86-1.21)	0.950	0.736	1.16(0.72-1.87)	0.950	0.063	1.02(0.89-1.19)	0.950	0.488
Consistent to HWE	7	1.03(0.70-1.51)	0.897	0.176	1.09(0.88-1.37)	0.430	0.844	1.09(0.89-1.35)	0.405	0.877	1.00(0.60-1.68)	0.998	0.082	1.06(0.89-1.22)	0.895	0.467
European	3	0.85(0.48-1.52)	0.747	0.783	1.20(0.85-1.70)	0.608	0.500	1.13(0.81-1.57)	0.727	0.453	0.79(0.46-1.36)	0.637	0.891	1.02(0.80-1.30)	0.892	0.526
Asian	4	1.10(0.62-1.93)	0.747	0.128	1.06(0.81-1.38)	0.690	0.960	1.07(0.83-1.38)	0.727	0.858	1.13(0.47-2.72)	0.786	0.103	1.04(0.85-1.27)	0.892	0.416
**OAT[c]**	10	1.09(0.84-1.40)	0.838	0.105	0.94(0.81-1.09)	0.838	0.169	0.98(0.81-1.18)	0.838	0.090	1.12(0.88-1.42)	0.838	0.241	1.01(0.88-1.17)	0.838	0.087
Consistent to HWE	9	1.01(0.77-1.32)	0.957	0.177	1.01(0.86-1.19)	0.916	0.285	1.02(0.83-1.25)	0.886	0.108	1.04(0.81-1.33)	0.768	0.495	1.03(0.87-1.21)	0.729	0.064
European	3	0.84(0.50-1.40)	0.610	0.842	0.76(0.54-1.06)	0.139	0.919	0.78(0.57-1.06)	0.207	0.915	0.96(0.59-1.56)	0.855	0.843	0.85(0.67-1.08)	0.386	0.890
Asian	5	0.91(0.65-1.29)	0.610	0.426	1.04(0.85-1.28)	0.698	0.463	1.04(0.83-1.29)	0.739	0.304	0.95(0.69-1.29)	0.855	0.677	1.02(0.86-1.21)	0.854	0.265

[a]Number of studies.

[b]The value of heterogeneity test.

[c]Including OAT, severe OAT, oligozoospermia, and teratozoospermia.

[d]p value in multiple testing (Benjamini-Hochberg methods).

Supplemental Table 3. Main results for the MTRR A66G mutation in the meta-analysis

MTRR A66G(rs1801394)		GG vs AA			AG vs AA			AG/GG vs AA			GG vs AG/AA			G vs A		
**Variables**	**N[a]**	**OR(95%CI)**	**P[b]**	**FDR[d]**	**OR(95%CI)**	**P[b]**	**FDR[d]**	**OR(95%CI)**	**P[b]**	**FDR[d]**	**OR(95%CI)**	**P[b]**	**FDR[d]**	**OR(95%CI)**	**P[b]**	**FDR[d]**
**Total**	7	1.24(0.98-1.58)	0.450	0.180	1.12(0.93-1.36)	0.633	0.230	1.16(0.97-1.40)	0.588	0.180	1.14(0.94-1.38)	0.492	0.230	1.10(0.99-1.23)	0.528	0.180
**Consistent to HWE**	5	1.15(0.87-1.51)	0.418	0.326	1.06(0.84-1.33)	0.525	0.646	1.10(0.88-1.36)	0.457	0.406	1.09(0.88-1.35)	0.427	0.439	1.07(0.94-1.22)	0.341	0.327
**Ethnicity**																
European	4	0.98(0.71-1.36)	0.853	0.894	0.96(0.72-1.27)	0.688	0.768	0.97(0.75-1.27)	0.721	0.844	0.99(0.78-1.27)	0.717	0.951	0.99(0.85-1.15)	0.824	0.880
Asian	2	1.61(1.04-2.50)	0.582	0.102	1.33(0.98-1.81)	0.609	0.189	1.37(1.02-1.84)	0.743	0.111	1.34(0.91-1.97)	0.462	0.212	1.20(1.00-1.43)	0.828	0.110
**Subgroup**																
**Azoospermia**	4	0.83(0.56-1.22)	0.390	0.594	0.89(0.66-1.22)	0.916	0.594	0.89(0.66-1.19)	0.803	0.594	0.88(0.65-1.18)	0.325	0.594	0.92(0.78-1.09)	0.539	0.887
Consistent to HWE	3	1.00(0.62-1.60)	0.576	0.995	0.96(0.64-1.46)	0.891	0.863	0.98(0.66-1.46)	0.825	0.932	0.97(0.69-1.38)	0.35	0.883	0.98(0.79-1.23)	0.492	0.350
**OAT[c]**	4	1.36(0.95-1.94)	0.299	0.217	1.09(0.80-1.48)	0.562	0.596	1.17(0.87-1.58)	0.478	0.369	1.24(0.95-1.61)	0.325	0.217	1.13(0.96-1.33)	0.344	0.217
Consistent to HWE	3	1.24(0.81-1.91)	0.208	0.320	0.99(0.66-1.46)	0.492	0.941	1.09(0.75-1.58)	0.359	0.654	1.19(0.87-1.62)	0.195	0.274	1.11(0.90-1.36)	0.199	0.332

[a]Number of studies.

[b]The value of heterogeneity test.

[c]Including OAT, severe OAT, oligozoospermia, and teratozoospermia.

[d]p value in multiple testing (Benjamini-Hochberg methods).
